# Dynamic changes and associated factors of asymmetric prominent vessel sign in atherosclerotic anterior circulation acute ischemic stroke: a preliminary study

**DOI:** 10.3389/fneur.2026.1868578

**Published:** 2026-07-01

**Authors:** Wenjun Li, Cuicui Liu, Jinyang Wang, Yanan Jia, Huiling Ren, Junyan Liu, Yuzhu Xu

**Affiliations:** Department of Neurology, Hebei Medical University Third Hospital, Shijiazhuang, China

**Keywords:** acute ischemic stroke, asymmetric prominent vessel sign, cerebral hemodynamics, multimodal imaging, susceptibility-weighted imaging

## Abstract

**Objective:**

To investigate the dynamic changes, influencing factors, and prognostic significance of the asymmetric prominent vessel sign (APVS) in patients with atherosclerotic anterior circulation acute ischemic stroke (AIS).

**Methods:**

Patients with atherosclerotic anterior circulation AIS who were admitted within 48 h after symptom onset and had complete baseline and follow-up diffusion-weighted imaging (DWI) and susceptibility-weighted imaging (SWI) data obtained 10–14 days after treatment were retrospectively enrolled. Changes in APVS were evaluated. Clinical and imaging data were collected, including the degree of vascular stenosis, baseline SWI–DWI mismatch, NIHSS scores before and after treatment, infarct volume, SWI-Alberta Stroke Program Early CT Score (SWI-ASPECTS), early neurological deterioration (END) within 7 days, and 90-day mRS scores. According to the difference in SWI-ASPECTS scores before and after treatment, patients were categorized into the significant APVS change group (≥2 points) and the non-significant APVS change group (0–1 point). Univariate analyses were performed to preliminarily explore the factors associated with APVS changes and the relationship between APVS changes and clinical outcomes.

**Results:**

A total of 34 patients were included, among whom 11 were assigned to the significant APVS change group. After 10–14 days of standardized conservative medical treatment, the APVS extent remained unchanged in 38.2% of patients, decreased to varying degrees in 58.8% (including complete disappearance in 17.6%), and increased in only 2.9% of patients. Univariate analysis demonstrated that patients in the significant change group had larger baseline infarct volumes (*T* = −2.191, *p* = 0.028) and post-treatment infarct volumes (*T* = −2.779, *p* = 0.005), a higher proportion of severe vascular stenosis (*p* = 0.009), and higher baseline SWI-ASPECTS scores (*T* = −3.117, *p* = 0.002). No statistically significant differences were observed in the incidence of END or 90-day mRS scores between the two groups.

**Conclusion:**

Following standardized treatment for 10–14 days, APVS extent decreased or even disappeared in most patients, whereas persistent APVS was observed in a subset of patients. Baseline and post-treatment infarct volumes, degree of vascular stenosis, and baseline SWI-ASPECTS scores may influence APVS evolution. However, APVS changes were not significantly associated with clinical prognosis.

## 1 Introduction

Ischemic stroke, characterized by high morbidity, disability, and mortality rates, poses a substantial threat to public health and has become a major global healthcare burden. With the continuous advancement of medical imaging technologies, the early diagnosis and precision management of ischemic stroke have markedly improved. Susceptibility-weighted imaging (SWI), which is based on the blood oxygen level-dependent effect, uses deoxyhemoglobin as an endogenous contrast agent to generate images by detecting differences in magnetic susceptibility among tissues. Owing to its high sensitivity to small veins and blood metabolic products, SWI has become an important neuroimaging technique in cerebrovascular diseases (1). In patients with acute ischemic stroke (AIS), SWI can not only detect cerebral microbleeds and newly formed red thrombi within arteries, thereby helping predict hemorrhagic transformation and vascular recanalization, but can also clearly demonstrate the asymmetrical prominent vessel sign (APVS).

The mechanisms underlying APVS remain incompletely understood, and two major hypotheses have been proposed. First, compensatory vasodilation under ischemic conditions may increase vascular volume, resulting in abnormally prominent vessels on SWI sequences. Second, ischemic regions exhibit an imbalance between oxygen supply and demand, leading to elevated deoxyhemoglobin levels and reduced oxyhemoglobin concentrations, which subsequently decrease intravascular signal intensity and create marked contrast relative to normal brain tissue (2). Previous studies have demonstrated that APVS is more frequently observed in AIS patients with anterior circulation involvement, severe vascular stenosis, or cardioembolic stroke (3). Furthermore, APVS has been reported to predict early neurological deterioration after stroke (4–7) and functional outcomes at 3 months after onset (8, 9). When combined with DWI, APVS may also facilitate the evaluation of collateral circulation status and the efficacy of reperfusion therapy (2, 10). In addition, accumulating evidence suggests that APVS is closely associated with the time from stroke onset, being most pronounced within the first several hours after acute cerebral infarction, gradually diminishing after 48 h, and potentially returning to a level comparable to that of the contralateral normal vessels by 12 weeks (11). However, despite increasing interest in APVS, studies focusing specifically on its dynamic evolution remain limited. Therefore, the present study enrolled patients with atherosclerotic anterior circulation AIS to preliminarily investigate the temporal changes in APVS, the factors influencing these changes, and their association with clinical prognosis.

## 2 Materials and methods

### 2.1 Study population

This retrospective study consecutively enrolled patients with acute anterior circulation infarction who were admitted to the Department of Neurology of the Third Hospital of Hebei Medical University between March 2022 and June 2025. The inclusion criteria were as follows: (1) age ≥18 years; (2) admission within 48 h after symptom onset; (3) pre-stroke modified Rankin Scale (mRS) score ≤1; (4) large artery atherosclerosis subtype according to the Chinese Ischemic Stroke Subclassification (CISS) criteria (12); (5) completion of baseline and follow-up neuroimaging examinations, including diffusion-weighted imaging (DWI) and SWI, at 10–14 days after treatment; (6) receipt of standardized conservative medical treatment after admission; and (7) complete clinical and imaging data available for analysis.

The exclusion criteria were as follows: (1) posterior circulation infarction; (2) non-atherosclerotic etiologies, including cardioembolic stroke and vasculitis; (3) prior reperfusion therapy; (4) severe bilateral intracranial or extracranial vascular stenosis; (5) poor image quality or significant imaging artifacts precluding radiological analysis; and (6) incomplete clinical data or duplicate hospitalization records.

### 2.2 Data collection and grouping

Demographic characteristics and clinical data of the enrolled patients were collected, including histories of hypertension, diabetes mellitus, coronary artery disease, smoking (defined as a previous long-term smoking history with smoking cessation for less than 1 year), and alcohol consumption (defined as a previous long-term drinking history with alcohol abstinence for less than 1 year). Laboratory parameters, including the blood lipid profile, blood glucose, C-reactive protein (CRP), homocysteine, and hemoglobin levels, were also recorded. In addition, the degree of vascular stenosis, baseline SWI–DWI mismatch status, NIHSS scores before and after treatment, infarct volume, SWI-Alberta Stroke Program Early CT Score (SWI-ASPECTS), occurrence of early neurological deterioration (END) within 7 days, and 90-day mRS scores were collected.

Patients were grouped according to the difference between pre-treatment and post-treatment SWI-ASPECTS scores. As no universally accepted cutoff value for SWI-ASPECTS score changes currently exists, grouping in the present study was based on the actual distribution characteristics of score differences within this cohort. Patients with score differences of 0 or 1 were assigned to the non-significant change group, whereas those with score differences ≥2 were assigned to the significant change group.

### 2.3 MRI equipment and imaging parameters

All cranial MRI examinations were performed using a 3.0-T MRI scanner (INGENIA 3.0 T CX; Philips Healthcare, the Netherlands). The imaging protocol included conventional MRI sequences, including T1-weighted imaging (T1WI), T2-weighted imaging (T2WI), fluid-attenuated inversion recovery (FLAIR), diffusion-weighted imaging (DWI), susceptibility-weighted imaging (SWI), and three-dimensional time-of-flight magnetic resonance angiography (3D TOF-MRA). DWI and SWI served as the primary sequences for imaging analysis in the present study.

The SWI sequence was acquired using a T2*-weighted imaging protocol with the following parameters: repetition time/echo time (TR/TE) = 31/7.2 ms, field of view = 23.0 cm × 18.9 cm, matrix size = 384 × 315, slice thickness = 2.5 mm, interslice gap = 1.0 mm, and acquisition time = 136 s. The SWI dataset included phase images, magnitude images, and minimum intensity projection (minIP) images.

### 2.4 Imaging analysis

#### 2.4.1 Assessment of APVS in ischemic regions

**Figure 1:**
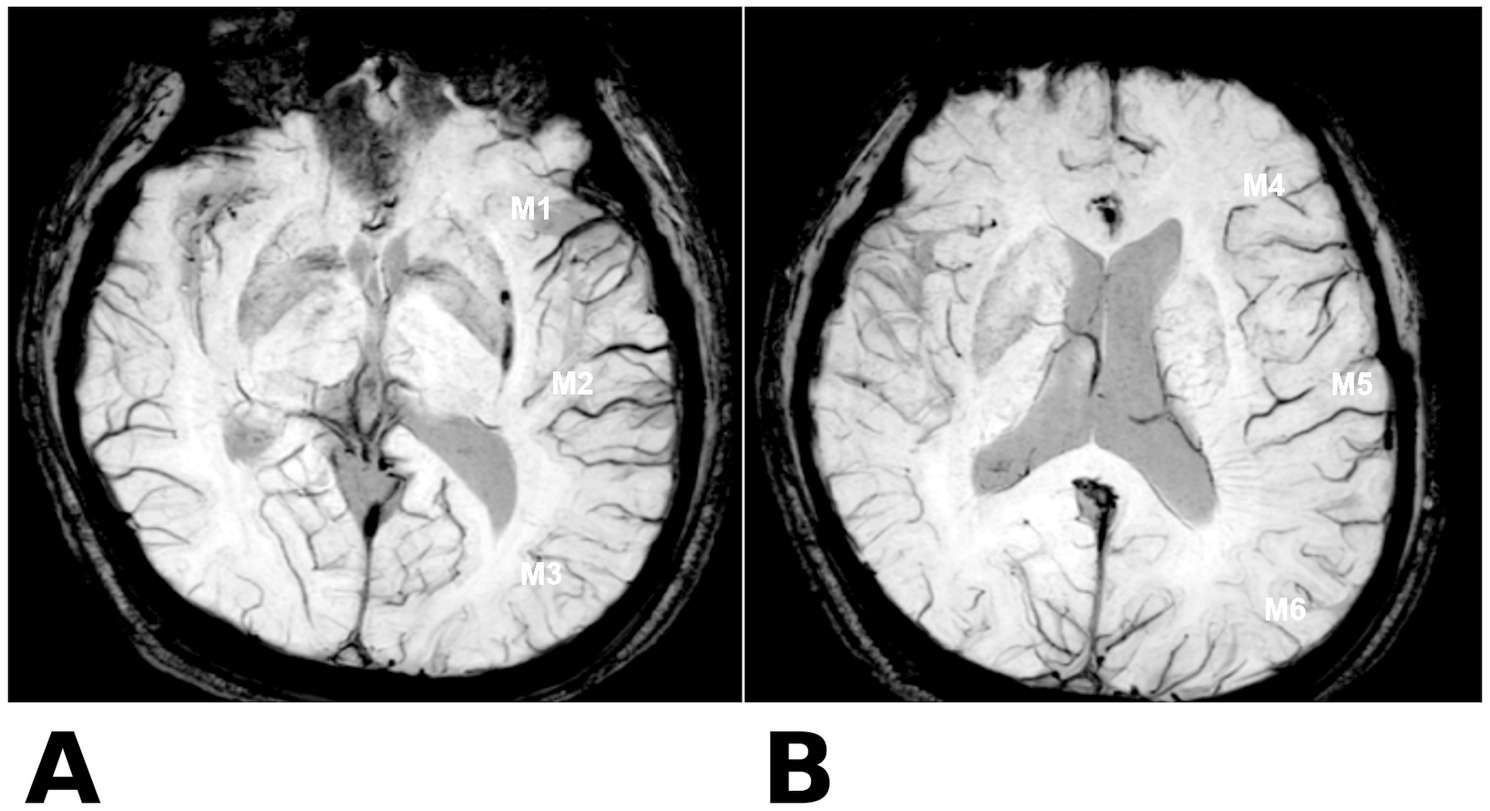
Definition and scoring of the APVS. **(A)** SWI showing lesions in regions M1–M3. **(B)** SWI showing lesions in regions M4–M6. The veins in the left cerebral hemisphere show an increased number and enlarged diameter compared with those in the contralateral hemisphere, indicating APVS positivity on susceptibility-weighted imaging (SWI). Panels **(A)** and **(B)** illustrate the anatomical regions used for scoring, including M1–M6 and the deep white matter. In this patient, APVS was observed in regions M1–M6, resulting in an SWI-ASPECTS score of 6.

Imaging interpretation SWI images were evaluated for the presence and distribution of APVS. APVS was defined as an increased number or diameter of hypointense venous structures in the affected hemisphere compared with the contralateral hemisphere on SWI. The extent of APVS was assessed using a modified 7-region SWI-ASPECTS scoring system based on previously reported SWI-ASPECTS/ACVS-ASPECTS regional assessment methods. In previous ACVS-ASPECTS approaches, the MCA territory was divided into eight regions, including the insula, deep white matter region, and M1–M6 cortical regions (13). In the present study, the insular region was excluded because APVS assessment in this area may be influenced by adjacent venous structures around the Sylvian fissure and partial-volume effects. Therefore, seven regions were assessed: M1, M2, M3, M4, M5, M6, and the deep white matter region. M1, M2, and M3 represented the anterior, lateral-to-insular, and posterior cortical MCA territories at the basal ganglia level, respectively; M4, M5, and M6 represented the anterior, lateral, and posterior cortical MCA territories above the basal ganglia level, respectively; and the deep white matter region included the basal ganglia and periventricular white matter. The presence of APVS in each region was assigned 1 point, yielding a total modified SWI-ASPECTS score ranging from 0 to 7. A higher score indicated a wider distribution of APVS (Figure 1).

#### 2.4.2 Infarct volume measurement

Baseline and post-treatment infarct volume was calculated on DWI images using the simplified Pullicino formula as follows: volume (cm^3^) = maximal lesion length × maximal lesion width × slice thickness × number of slices / 2. The MRI slice thickness in this study was 0.5 cm, thus the calculation formula was volume (cm^3^) = maximal lesion length × maximal lesion width × 0.5 × number of slices / 2. For patients with multiple lesions, the total infarct volume was calculated as the sum of the volumes of all newly identified lesions.

#### 2.4.3 Evaluation of culprit vessel stenosis

The degree of culprit arterial stenosis was calculated according to previously published methods (14). Mild-to-moderate stenosis was defined as a stenosis rate <70%, whereas severe stenosis was defined as a stenosis rate ≥70%.

#### 2.4.4 Assessment of SWI–DWI mismatch

SWI–DWI mismatch was defined as the extent of hypointense vascular signals observed on SWI exceeding the area of diffusion restriction identified on DWI (15).

All neuroimaging data were independently and blindly evaluated by two neurologists specializing in cerebrovascular diseases. The mean intraclass correlation coefficient (ICC) for pre-treatment and post-treatment SWI-ASPECTS scores was 0.933 (95% confidence interval [CI]: 0.866–0.967), indicating excellent interobserver agreement.

### 2.5 Definition of early neurological deterioration

END was defined as an increase of ≥2 points in the total NIHSS score or an increase of ≥1 point in the motor subscore within 7 days after admission (16).

### 2.6 Prognostic follow-up

Clinical follow-up was conducted by telephone at 3 months ± 7 days after stroke onset. mRS scores were recorded for all enrolled patients. An mRS score ≤1 was defined as a favorable outcome, whereas an mRS score of 2–5 was considered a poor outcome; a score of 6 indicated death.

### 2.7 Statistical analysis

Statistical analyses were performed using SPSS version 25.0 (IBM Corp., Armonk, NY, United States). Normally distributed continuous variables were expressed as the mean ± standard deviation (SD), and comparisons between groups were conducted using the independent-samples *t*-test. Non-normally distributed continuous variables were expressed as the median and interquartile range (IQR), and intergroup comparisons were performed using nonparametric tests. Categorical variables were presented as frequencies and compared using Fisher’s exact test. A two-sided *p*-value <0.05 was considered statistically significant.

## 3 Results

**Figure 2:**
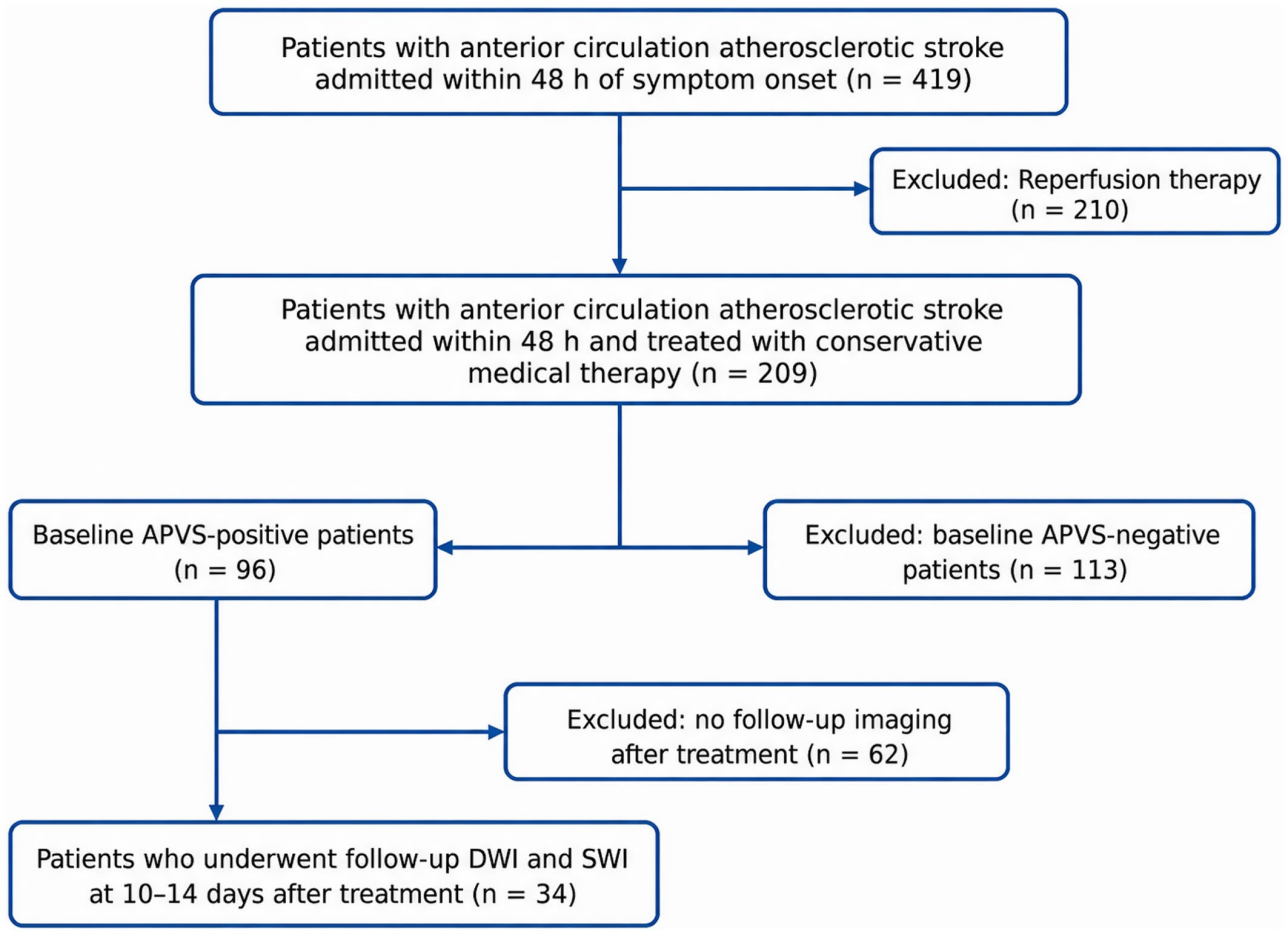
Flow chart of participant inclusion and exclusion.

A total of 34 patients met the inclusion criteria and were enrolled in the present study, including 26 men (76.5%), with a mean age of 62.9 ± 13.1 years (Figure 2). The median baseline SWI-ASPECTS score was 3, whereas the median post-treatment SWI-ASPECTS score was 2. Follow-up SWI examinations after treatment demonstrated that the extent of APVS remained unchanged compared with baseline in 13 patients (38.2%). In contrast, 20 patients (58.8%) exhibited varying degrees of reduction in SWI-ASPECTS scores after treatment, among whom APVS completely disappeared in 6 patients (17.6%) (Figure 2). Only 1 patient (2.9%) showed an increased SWI-ASPECTS score after treatment relative to baseline. According to the difference between pre-treatment and post-treatment SWI-ASPECTS scores, patients were categorized into the non-significant change group (score difference of 0–1; *n* = 23) and the significant change group (score difference ≥2; *n* = 11).

Univariate analysis demonstrated no significant differences between the two groups in demographic characteristics, medical histories, hematological parameters, baseline and post-treatment NIHSS scores, SWI–DWI mismatch status, the occurrence of END within 7 days, or 3-month mRS scores. However, patients in the significant change group exhibited larger baseline infarct volumes (P=0.028) and larger post-treatment infarct volumes (P=0.005) , a higher prevalence of severe vascular stenosis (P=0.009) , and higher baseline SWI-ASPECTS scores (P=0.002) compared with those in the non-significant change group (Table 1).

**Table 1:** Clinical and imaging characteristics associated with changes in the asymmetric prominent vessel sign (APVS).

Variables	Non-significant APVS change (*n* = 23)	Significant APVS change (*n* = 11)	Statistic	*p*-value
Male sex, *n* (%)	17 (73.9%)	9 (81.8%)	–	1.000
Age, years	64.5 ± 12.6	59.6 ± 14.0	1.03	0.311
Body mass index, kg/m^2^	26.4 ± 3.7	26.4 ± 4.4	0.04	0.971
Mean arterial pressure, mmHg	109.3 (100.7, 120)	101 (97.7, 124.7)	−0.39	0.699
History of hypertension, *n* (%)	13 (56.5%)	8 (72.7%)	–	0.465
History of diabetes mellitus, *n* (%)	6 (26.1%)	2 (18.2%)	–	1.000
History of smoking, *n* (%)	10 (43.5%)	5 (45.5%)	–	1.000
History of alcohol consumption, *n* (%)	8 (34.8%)	5 (45.5%)	–	0.709
Coronary heart disease, *n* (%)	3 (13.0%)	2 (18.2%)	–	1.000
Hemoglobin, g/L	142.8 ± 15.6	133.6 ± 15.0	1.62	0.116
Fibrinogen, g/L	2.9 (2.6, 3.7)	2.8 (2.3, 3.2)	−1.29	0.198
LDL cholesterol, mmol/L	2.8 ± 0.7	2.5 ± 0.8	1.42	0.167
HDL cholesterol, mmol/L	1.1 ± 0.2	1.1 ± 0.4	−0.12	0.908
Total cholesterol, mmol/L	4.3 ± 1.2	4.2 ± 0.7	0.45	0.655
Triglycerides, mmol/L	1.4 (1.1, 1.7)	1.2 (0.8, 1.2)	−1.27	0.204
Blood glucose, mmol/L	6.5 (5.1, 8.1)	6.2 (5.7, 8.6)	−0.57	0.568
Homocysteine, μmol/L	14.0 (13.1, 16.9)	13.3 (10.1, 17.4)	−0.72	0.473
Uric acid, μmol/L	346.7 ± 104.7	363.7 ± 119.2	−0.43	0.673
Glycated hemoglobin, %	5.9 (5.6, 6.6)	5.8 (5.5, 6.1)	−0.46	0.644
Admission NIHSS score	2 (1, 4)	4 (2, 4)	−0.88	0.378
Discharge NIHSS score	2 (1, 4)	3 (1, 5)	−0.96	0.338
Baseline infarct volume, cm^3^	0.4 (0.2, 1.9)	1.5 (1.0, 6.7)	−2.19	0.028
Post-treatment infarct volume, cm^3^	0.5 (0.1, 1.4)	2.8 (0.9, 12.0)	−2.78	0.005
Infarct volume change (post − pre), cm^3^	−0.3 (−0.4, 0.6)	0.18 (−0.2, 0.18)	−1.12	0.262
Severe arterial stenosis, *n* (%)	7 (30.4%)	9 (81.8%)	–	0.009
SWI-DWI mismatch, *n* (%)	17 (73.9%)	9 (81.8%)	0.01	0.939
Baseline SWI-ASPECTS score	3 (2, 4)	5 (4, 6)	−3.12	0.002
Post-treatment SWI-ASPECTS score	2 (1, 3)	1 (0, 3)	−1.41	0.158
END, *n* (%)	5 (21.7%)	4 (36.4%)	–	0.452
Favorable outcome (mRS > 1), *n* (%)	5 (21.7%)	4 (36.4%)	–	0.452

APVS, asymmetric prominent vessel sign; BMI, body mass index; NIHSS, National Institutes of Health Stroke Scale; LDL, low-density lipoprotein; HDL, high-density lipoprotein; DWI, diffusion-weighted imaging; SWI, susceptibility-weighted imaging; ASPECTS, Alberta Stroke Program Early CT Score; mRS, modified Rankin Scale.

## 4 Discussion

**Figure 3:**
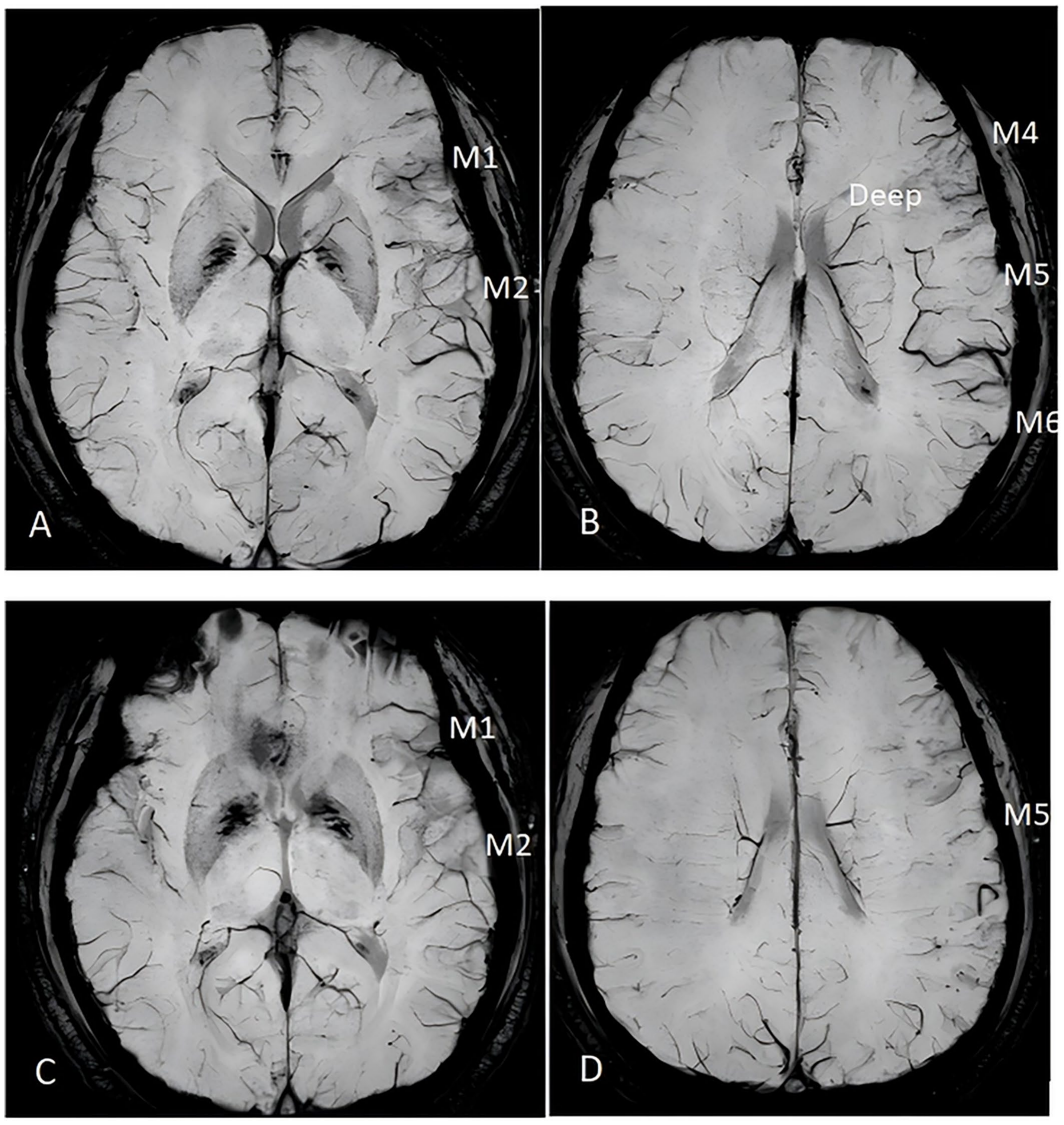
Representative case illustrating dynamic changes in APVS. A 48-year-old man presented with episodic dysarthria and impaired responsiveness for 10 h. Baseline SWI **(A,B)** demonstrated an SWI-ASPECTS score of 6, with APVS involving the M1–M5 cortical regions and deep structures. Follow-up SWI after 10 days of standardized medical management **(C,D)** showed a decreased SWI-ASPECTS score of 3, with APVS remaining in the M1, M2, and M5 regions.

The present study demonstrated that after 10–14 days of standardized conservative medical treatment, the extent of APVS decreased to varying degrees in 58.8% of enrolled patients, with complete disappearance observed in 17.6% of cases. These findings suggest that, in most patients, the extent of APVS gradually diminishes or even completely resolves with disease progression and the implementation of standardized therapeutic interventions. A previous study using quantitative susceptibility mapping (QSM) reported that among 39 patients with baseline APVS positivity, APVS completely disappeared in 35 patients during follow-up at 2 weeks after treatment (17). Along with the APVS resolution, venous oxygen saturation (SvO₂) significantly increased, and the degree of SvO₂ improvement was positively correlated with clinical outcomes. The authors proposed that the dynamic evolution of APVS may effectively reflect cerebral oxygen utilization and metabolic regulation following acute ischemic stroke. Furthermore, the disappearance of APVS was considered attributable to improvements in cerebral perfusion and neurological function after treatment, which could be indirectly reflected by changes in tissue oxygen extraction capacity. From a pathophysiological perspective, the presence of APVS indicates a state of cerebral hypoperfusion, during which ischemic brain tissue activates compensatory metabolic reserves to maintain cellular viability. Therefore, APVS may partially represent an imaging manifestation of the ischemic penumbra. However, the ischemic penumbra is a dynamic rather than permanent condition and may subsequently evolve toward two different outcomes: progression into infarct core tissue or salvage following restoration of cerebral perfusion. Both processes may ultimately result in a reduction or complete disappearance of APVS on follow-up imaging (Figure 3).

The present study further demonstrated that the extent of APVS remained largely unchanged in 38.2% of patients after treatment. Previous studies have also reported the presence of APVS in patients with asymptomatic intracranial arterial stenosis (18, 19). Del Poggio et al. (18) observed APVS in 22 of 37 patients with asymptomatic chronic intracranial or extracranial anterior circulation stenosis/occlusion. The authors suggested that APVS was associated with chronic cerebral hypoperfusion, impaired tissue perfusion, and collateral circulation formation, indicating that APVS may help identify patients who could potentially benefit from revascularization therapy. Collectively, these findings indicate that APVS is not restricted to the acute phase of stroke; rather, its presence fundamentally reflects a state of regional cerebral hypoperfusion. Previous studies have demonstrated a strong association between APVS and reduced cerebral blood flow accompanied by increased cerebral blood volume (10). Persistent APVS under such conditions may primarily result from compensatory vasodilation in response to ischemia, leading to increased vascular capacity and representing a benign oligemic state that may persist for a prolonged period. In addition, previous investigations have reported that the risk of APVS increases with the severity of white matter injury (20). Whether patients with persistent APVS are at greater risk of developing white matter damage or ischemic cerebral atrophy warrants further investigation in larger prospective studies.

In the present study, patients were categorized into the significant APVS change group and the non-significant APVS change group according to differences in SWI-ASPECTS scores before and after treatment. Univariate analysis was subsequently performed to preliminarily explore the factors influencing APVS evolution. The results demonstrated that patients with larger infarct volumes, more severe vascular stenosis, and higher baseline SWI-ASPECTS scores tended to exhibit more pronounced changes in APVS scores. Infarct volume and the severity of vascular stenosis are known to influence APVS visualization. In addition, the impact of baseline SWI-ASPECTS scores on subsequent APVS score changes may inevitably be affected by ceiling and floor effects. However, owing to the relatively limited sample size in the present study, multivariate analyses could not be performed to further adjust for potential confounding factors and identify independent predictors. Moreover, stratified analyses based on baseline SWI-ASPECTS scores were not feasible. Therefore, the present findings should be interpreted with caution. Future studies with larger sample sizes are warranted to validate these observations through comprehensive multivariate and stratified analyses.

Previous studies have demonstrated that the presence, extent, and distribution of APVS are associated with END (4–7), and that SWI–DWI mismatch may serve as an independent predictor of END (21). In addition, APVS has been reported to be associated with 90-day functional outcomes after stroke (8, 9). Based on these findings, the present study further explored the relationships between dynamic APVS changes before and after treatment and both END and 90-day functional outcomes. However, our results showed that APVS evolution was not significantly associated with either END or 90-day mRS scores. In addition, the proportion of patients with SWI–DWI mismatch did not differ significantly between the two groups. Previous studies have suggested that SWI–DWI mismatch may serve as a noninvasive imaging marker for indirectly evaluating collateral compensation and the presence of potentially salvageable ischemic penumbra (22). Because clinical outcomes in acute ischemic stroke are closely related to the duration of penumbral viability and the status of collateral circulation, the absence of a significant difference in SWI–DWI mismatch between groups may partly explain why dynamic APVS changes were not significantly associated with clinical prognosis in the present study. Several additional factors may also account for this finding. First, after standardized conservative medical treatment, APVS extent decreased substantially or remained stable in most patients. However, a reduction or disappearance of APVS may have different clinical implications. It may indicate reperfusion and subsequent neurological improvement, but it may also reflect progression of local ischemia with infarct evolution. Similarly, persistently unchanged APVS after treatment may be associated with heterogeneous clinical outcomes. Therefore, dynamic APVS changes alone may not be sufficient to reliably distinguish favorable from unfavorable clinical trajectories. Second, although SWI–DWI mismatch has been proposed as a surrogate marker of ischemic penumbra and tissue hypoperfusion, it may primarily reflect the hemodynamic status during the acute phase of stroke rather than the subsequent dynamic evolution of APVS during follow-up. Third, the relatively small sample size and the high incidence of SWI–DWI mismatch in both groups may have limited the statistical power to detect significant associations. Taken together, these findings suggest that dynamic APVS changes alone may not be sufficient to reliably predict disease progression or long-term functional outcomes in acute ischemic stroke. Nevertheless, these conclusions should be interpreted with caution because of the limited sample size and relatively short observation period. Further large-scale prospective studies with extended follow-up are warranted to validate these findings.

Several limitations of this study should be acknowledged. First, this was a single-center retrospective study with a relatively small sample size. The enrolled patients generally had mild neurological deficits, which may have introduced potential selection bias. In addition, residual confounding could not be fully excluded, thereby limiting the representativeness and generalizability of the findings. Second, the method used for infarct volume measurement in this study had limited precision. Compared with fully automated imaging segmentation techniques, this approach may be more susceptible to measurement error, particularly for irregular or non-elliptical infarcts, which may have affected the accuracy of the correlation analyses. This modified regional scoring system has not yet been validated in an external cohort. Moreover, no universally accepted standardized SWI-ASPECTS scoring system is currently available, and substantial heterogeneity exists across studies in terms of regional classification and scoring criteria. This limits direct comparisons between studies. Future multicenter prospective studies with larger sample sizes are warranted. The application of high-precision, fully automated imaging segmentation techniques may help validate the reliability and validity of this modified SWI-ASPECTS regional scoring system and further strengthen the conclusions of the present study.

## 5 Conclusion

This multimodal data-driven study demonstrated that APVS decreased or disappeared in most patients with atherosclerotic anterior circulation AIS after standardized conservative treatment, although persistence was observed in some cases. Infarct volume, vascular stenosis severity, and baseline SWI-ASPECTS score may influence APVS evolution, whereas APVS dynamics were not significantly associated with clinical prognosis. Larger prospective studies are needed to validate these findings.

## Data Availability

The original contributions presented in the study are included in the article/supplementary material, further inquiries can be directed to the corresponding author.
